# Lactate as a regulator of iron homeostasis

**DOI:** 10.1093/lifemeta/load033

**Published:** 2023-07-27

**Authors:** Gregory J Anderson, David M Frazer

**Affiliations:** Iron Metabolism Laboratory, QIMR Berghofer Medical Research Institute, Brisbane, Queensland 4006, Australia; Molecular Nutrition Laboratory, QIMR Berghofer Medical Research Institute, Brisbane, Queensland 4006, Australia

**The key metabolic intermediate lactate can increase expression of the liver-derived peptide hepcidin, the central regulator of body iron homeostasis. A new paper by Liu *et al*. shows that lactate achieves this by binding to and activating soluble adenylyl cyclase, thereby increasing cellular cyclic adenosine monophosphate (cAMP) and enhancing signaling through the bone morphogenetic protein (BMP) pathway to modulate hepcidin expression.**

Iron is an essential trace element and a large number of metabolic processes are dependent on iron for their normal functions. The chemical plasticity of iron makes it ideally suited to electron transfer, and many proteins use iron at their active centers in the form of haem, iron-sulfur clusters, and a wide range of other conformations. Indeed, there are very few organisms that do not use iron in this way and most life forms have an absolute requirement for iron. In mammals, most iron is used for haemoglobin production in developing red blood cells, but the much smaller amount that is directed to myriad other proteins in all cells is no less critical.

The same catalytic qualities that make iron essential also mean that it can be detrimental, as iron can promote reactions leading to the generation of toxic oxygen radicals. To keep the balance between essentiality and toxicity in check, iron levels must be kept within a defined physiological range, both at the cellular and whole-body levels. Critical for this regulation is the ­liver-derived peptide hormone hepcidin [1]. Often called the master regulator of body iron homeostasis, hepcidin expression responds to variations in body iron requirements to determine how many iron cells release. It does this by binding to the plasma membrane iron exporter ferroportin and targeting it for degradation. When body iron levels are low, hepcidin expression is reduced and ferroportin levels remain relatively high to enable iron to be exported from storage sites and directed to other sites in the body where it is required for metabolic functions. The opposite occurs under iron-replete conditions, where increased hepcidin acts to reduce ferroportin expression, thus decreasing iron mobilization from storage.

The most prominent factors controlling the expression of hepcidin are body iron levels, the rate of erythropoiesis, and inflammation, but many other factors have been shown to influence hepcidin production to varying degrees. A recent intriguing paper published by Liu and colleagues in *Cell Metabolism* [2] explores the mechanisms by which lactate can act as a regulator of hepcidin. Their studies were initially driven by the observation that physiological or pathological situations associated with high levels of lactate (such as vigorous physical activity, certain cancers, diabetes, and ischemia) are also often characterized by low iron levels and anemia. For example, the anemia associated with strenuous physical activity has been well recognized for many years, and a range of factors such as baseline iron status and low-level inflammation have been considered to contribute to this process [3], but now lactate must be considered to be involved in this exercise response as well. While a correlation between increased lactate and physical activity-associated anemia does not demonstrate a causative link, the new study presents an extensive series of experiments that provide convincing evidence that lactate plays an important role in driving this association by stimulating the expression of hepcidin. Although not the first study to show that lactate can drive hepcidin expression, it is certainly the most comprehensive.

Lactate was historically considered a waste product of glycolysis, produced via the fermentation of glucose under oxygen-poor conditions. However, even in the presence of oxygen, cells can readily produce lactate, as described by Warburg many years ago. The Warburg effect is typically considered a characteristic of cancer cells, but lactate can act as an important energy source more broadly, and it can also act as a feedback regulator of metabolic processes and as a signaling molecule [4, 5]. So, far from being a waste product, lactate is an important component of metabolism that mediates a network of both general and cell type-specific processes.

In their study, Liu and colleagues initially verified the association between increased lactate, hepcidin, and various ­iron-related parameters in a cohort of normal human volunteers, then followed this with studies in both exercising humans and mice. In the normal human cohort, a positive correlation between lactate and hepcidin was observed, but only at lactate concentrations above the upper limit of the normal range (i.e. approximately 1–2 mmol/L), suggesting that lactate concentrations need to be quite high if they are to influence hepcidin and iron levels. It was not clear in this cross-sectional study why some individuals had high lactate levels, so it is possible that the lactate and hepcidin changes were independently influenced by one or more additional factors. A more direct link was shown in the exercise studies, where human volunteers undertook three short periods of intense exercise with intervening rest periods. They were then studied for 20 min and 6 h after the final burst of activity, respectively. Lactate was increased several fold at the 20-min time point, but had returned to baseline by 6 h. This is not surprising as lactate is known to be cleared quite quickly from the circulation, with reported half-lives typically in the range of 10–20 min for healthy individuals after exercise [6]. The high-intensity exercise was also associated with an increase in the circulating hepcidin level and a concomitant reduction in serum iron. Interestingly, hepcidin expression was only marginally elevated immediately after exercise (in both humans and mice), but considerably increased after 6 h. This temporal discrepancy was not explored in the study, but earlier work by Goto *et al.* [7], with a broadly similar experimental design, showed that hepcidin peaked 3 h after the completion of resistance exercise. In the study of Goto *et al*., hepcidin remained elevated after 6 h, although the level had dropped to only about half the concentration reached at the 3-h peak. This raises the possibility that the peak in hepcidin expression may have been missed in the study of Liu *et al*., but remains consistent with the hepcidin peak occurring hours after the lactate peak. If lactate is a significant regulator of hepcidin expression, as extensive further studies by Liu *et al*. indicate (see below), further work is required to determine why the peak in hepcidin expression follows so long after the peak in lactate production.

Since hepcidin expression can be strongly increased by inflammation and vigorous exercise is often accompanied by an increase in pro-inflammatory cytokines, it has generally been considered that inflammation is a key factor responsible for increasing hepcidin expression post-exercise. Interestingly, however, Liu *et al*. did not find evidence of overt inflammation in their experimental cohort. The nature, intensity, and duration of the physical activity are important factors in determining the relative contribution of inflammation to hepcidin expression, so it is possible that the short bursts of intense exercise employed were insufficient to raise inflammatory markers in this study. It is also possible that there were some effects of low-level inflammation, but these effects were not strong enough to lead to elevated serum markers at the time points studied. Thus, these experiments strengthen the case for the role of lactate in altering hepcidin levels following exercise but do not exclude a contribution of inflammation.

Following the tantalizing results of their exercise studies, Liu *et al*. then carried out a more detailed analysis of the possible effects of lactate on hepcidin expression *in vitro*. These studies demonstrated that direct application of lactate to several cell lines increased the expression of the mRNA encoding hepcidin approximately 2-fold, whereas other metabolites (pyruvate, citrate, NADH) and a reduced pH had no effect. In a previous study, Mizumoto *et al*. also found that lactate treatment increased hepcidin expression in several cell lines, but in that study, they considered that the acidosis itself was important as hydrochloric acid and increased carbon dioxide exerted similar effects to lactate [8]. Liu *et al*. showed that high concentrations of lactate increased hepcidin expression quite quickly, although prolonged incubations were required for maximum effect, whereas lower concentrations took 6–12 h for an increase to be apparent. A fuller investigation of the mechanisms underlying these dose- and time-dependent changes would have been interesting, but these experiments provide further evidence that lactate is an important regulator of hepcidin.

**Figure 1:**
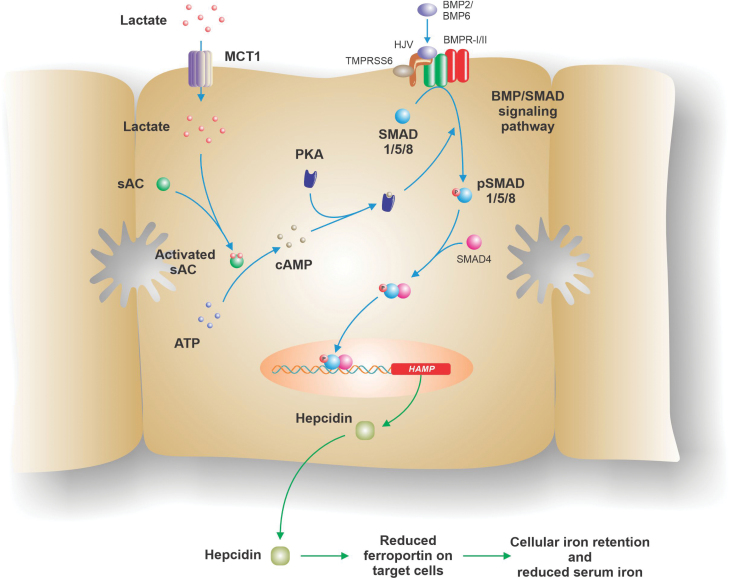
Proposed mechanism by which lactate stimulates hepcidin expression. Lactate enters cells (such as hepatocytes, as shown here) via the transporter MCT1 and subsequently binds to sAC, thereby activating the enzyme. sAC in turn converts ATP to cAMP, which signals via PKA to stimulate the activity of the SMAD signaling pathway. Enhanced SMAD signaling increases transcription of the *HAMP* gene, which encodes hepcidin, the master regulator of iron homeostasis. Hepcidin then binds to the iron export protein on the surface of target cells and the complex is internalized and degraded. This leads to iron retention in these cells and a reduction in circulating iron levels. BMP, bone morphogenetic protein; BMPR-I/II, bone morphogenetic protein type I or II receptor; cAMP, cyclic adenosine monophosphate; HJV, haemojuvelin; *HAMP*, hepcidin gene; MCT1, monocarboxylate transporter 1; PKA, protein kinase A; sAC, soluble adenylyl cyclase; SMAD, small mothers against decapentaplegic; TMPRSS6, transmembrane protease, serine 6, a type II transmembrane serine protease, also known as matriptase-2.

How does lactate increase hepcidin expression? Several signaling pathways are known to be involved in the regulation of hepcidin expression. The most significant of these are the bone morphogenetic protein (BMP)-small mothers against decapentaplegic (SMAD) and the Janus kinase-signal transducer and activator of transcription (JAK-STAT) pathways which play central roles in the regulation of hepcidin by iron and inflammation, respectively [1]. *In vitro* studies by Liu *et al*. demonstrated that the effects of lactate were mediated via the SMAD signaling. They found that phosphorylation of SMAD1/5/8, key SMAD signaling intermediates, was stimulated by lactate treatment, but phosphorylation of STAT3 was unaffected. Then they uncovered key details of the upstream processes that ultimately led to activation of the SMAD signaling by carrying out a detailed series of *in vitro* studies. The results of these studies are summarized as follows (and in Fig. 1):

1 Lactate enters cells via monocarboxylate transporter 1 (MCT1) and functional MCT1 is required for the hepcidin response.
2 The effects of lactate on hepcidin are independent of the signaling molecules G protein-coupled receptor 81 (GPR81) and GPR132, known sensors for lactate.
3 Lactate increases cellular levels of cyclic adenosine monophosphate (cAMP). An increase in the level of phosphorylated cAMP-responsive element binding protein 1 (CREB1) following lactate treatment was consistent with lactate-stimulating cAMP-mediated signaling, as was the demonstration that an inhibitor of protein kinase A (PKA), an important mediator of the effects of cAMP, blocked the effects of lactate on hepcidin. Importantly, inhibition of PKA also prevented the phosphorylation of SMAD1/5/8 in response to lactate. Forskolin, an activator of PKA, increased SMAD1/5/8 phosphorylation and the expression of hepcidin.
4 Lactate also increases the level of intracellular ATP and this may be the source of the cAMP that underlies its signaling. Consistent with this, ATP administration was shown to stimulate hepcidin expression via PKA activation, and the ATP synthase inhibitor oligomycin A reduced the lactate-mediated increases in both ATP and cAMP.
5 ATP is converted to cAMP by adenylyl cyclases, and the investigators showed that the effects of lactate on hepcidin can only be blocked when the soluble adenylyl cyclase (sAC) Adcy10 was knocked down.
6 The final piece of this puzzle is the demonstration that lactate directly binds to sAC. A crystal structure of the lactate/sCA complex was generated to examine the details of this binding, and, based on the structural details of the lactate/sAC interaction, a possible mechanism was proposed by which enzyme activity was stimulated.

These detailed studies clearly show how lactate increases hepcidin expression through the ATP/cAMP-mediated stimulation of the SMAD signaling pathway. However, many of the finer details of this process have yet to be revealed. In particular, the expression of hepcidin is modulated by many stimuli, some of which interact with the BMP-SMAD pathway and some of which do not. This means that the net synthesis of hepcidin is determined by many factors, but we do not yet have a comprehensive picture of the relative contributions of these various inputs under differing physiological and pathophysiological conditions. It seems likely that lactate levels are not a major determinant of hepcidin concentrations in healthy humans, but it certainly could assume a far more significant role under physiological or pathological stress.

The *in vitro* studies presented by Liu *et al*. enabled a considerable amount of detail about the mechanism of lactate action to be gleaned. However, are similar effects seen *in vivo*? This is an important consideration as some of the key aspects of hepcidin regulation have been proved difficult to replicate in cell culture studies, likely because, multiple cell types *in vivo* are involved in the full hepcidin regulatory pathway. Liu *et al*. addressed this by returning to mouse studies. The administration of lactate intravenously to mice on alternate days for one week led to an approximately 50% increase in hepcidin expression, and a concomitant decrease in serum iron and increase in splenic iron levels. (Interestingly, and unexpectedly, however, hepatic iron levels were decreased.) Lower small intestinal ferroportin expression in lactate-treated mice and an associated increase in iron levels in duodenal enterocytes were also consistent with lactate exerting a systemic effect on iron homeostasis. Associated studies showed that lactate treatment exacerbated anemia in iron-deficient mice and the recovery from iron deficiency. These experiments clearly showed that lactate can act as a driver of hepcidin change and this in turn influences body iron distribution. When analogous studies were carried out in mice lacking hepcidin, no effect of lactate on iron parameters was observed, indicating that its iron effects were mediated through hepcidin. Importantly, lactate treatment *in vivo* led to increased phosphorylation of CREB1 and SMAD1/5/8, and prior *in vivo* knockdown of sAC significantly reduced the degree to which lactate enhanced hepcidin expression. These studies are consistent with the cell culture studies that defined key elements in the mechanism of lactate action. The *in vivo* studies also showed that lactate administration did not alter the expression of BMP2 or BMP6, well-known upstream regulators of hepcidin via the SMAD signaling pathway in response to some stimuli such as altered iron levels. Thus, the influence of lactate on SMAD signaling is independent of at least these BMPs.

Any study demonstrating a novel pathway inevitably leads to a number of questions that need to be followed up in future work. One fundamental question is what is the physiological advantage of a lactate-stimulated increase in hepcidin? In their study, the authors speculated that an increase in hepcidin acts to facilitate cellular iron retention, thereby allowing increased catabolism of lactate. However, this seems unlikely given that lactate returns to normal after exercise well before there is a substantial increase in hepcidin expression. Also inconsistent with increased cellular iron being important for lactate catabolism is an earlier demonstration using exercising rats that severe iron deficiency leads to an increase in lactate production, with no change in lactate removal [9]. Furthermore, under such conditions, an increase in hepcidin expression would be detrimental as it would exacerbate the anemia. Additional studies would be very useful in helping to understand the basis of these discrepancies. More work also needs to be carried out to better understand the temporal relationships between changes in lactate levels and changes in hepcidin expression. Why does the peak in hepcidin expression occur hours after the peak in lactate? It would seem, based on what the cellular studies revealed about the mechanism of lactate action, that it should not take long for lactate to enter cells and exert its effects on the pathways that alter hepcidin expression, but, *in vivo*, this seems to be a relatively prolonged process. It would have been informative to determine how long it takes hepcidin expression to change after a single “burst” of lactate administered by injection. The investigators stated that a single injection of lactate can provide a substantive increase in hepcidin expression, but in their paper, they only presented data from studies using a course of lactate injections. Also worthy of further investigation is why the effects of lactate on hepcidin expression only appear to be notable at higher lactate concentrations. What is the basis of this threshold effect?

Overall, the studies presented by Liu *et al*. present a compelling case that lactate can act as a regulator of hepcidin, and it needs to be added to the inventory of factors that can influence the production of this major regulatory peptide. Important observations have been made on the mechanism by which lactate exerts its effects on hepcidin, and its ability to modulate SMAD signaling is consistent with the significance of this regulatory pathway for controlling hepcidin synthesis. Most of the other known modulators of hepcidin also act through this pathway. What is more difficult to assess is the relative importance of lactate as a hepcidin regulator. The effects of exogenous lactate administration on hepcidin expression observed in this study, both *in vivo* and *in vivo*, were relatively modest compared to other stimuli such as changes in iron levels, erythropoiesis, or inflammation. It is likely that under normal physiological conditions, the role of lactate is relatively small, but in situations characterized by elevated lactate, such as strenuous physical activity or some pathological conditions like lactatemia, lactate could assume a more prominent role.
